# Pertussis epidemiology and seroprevalence in Wujin District, Changzhou, China, 2024

**DOI:** 10.1186/s12879-026-12799-5

**Published:** 2026-02-12

**Authors:** Chunyan Yuan, Lingyan Tang, Cong Chen, Weiyi Zhuang, Hong Zhang, Qin Xu, Yingzi Pan

**Affiliations:** Department of Program on Immunization, Wujin District Center for Disease Control and Prevention, NO.2 Funan Rd, Wujin District, Changzhou, Jiangsu Province China

**Keywords:** Pertussis, Serological epidemiology, Healthy population

## Abstract

**Objectives:**

China experienced its worst pertussis epidemic in 30 years during 2024, and Wujin District faced the same challenge. This study aims to investigate the epidemiological characteristics of pertussis and the immune status of the healthy population in Wujin District, Changzhou City.

**Methods:**

The study employed descriptive epidemiology for pertussis case analysis and a serological survey for population antibody assessment in Wujin District, 2024. The level of pertussis toxin (PT) IgG antibody was measured by the Enzyme-Linked Immunosorbent Assay (ELISA).

**Results:**

A total of 28 pertussis cases were reported in 2024 in Wujin District, with an incidence rate of 2.15 per 100,000 population. Although infants < 1 year had the peak incidence (84.02 per 100,000), the 5–9 year age group accounted for the largest proportion of cases (67.86%) with an incidence rate of 28.70 per 100,000. 521 participants were included in the serological survey. Anti-PT IgG levels were undetectable (< 5 IU/ml) in 49.52% of the participants. The overall positivity (≥ 20 IU/mL) rate and antibody concentration were 17.27% and 5.17 IU/mL, respectively. The antibody positive rate was highest in the 1.5–3 years age group (30.00%) and lowest in the 20–29 years age group (7.89%). The recent infection (≥ 80 IU/mL) rate was 2.11%, with the top two observed in the 6–9 years old group (7.69%) and the 10–14 years old group (5.88%). The seropositivity rates and antibody concentrations of vaccinated individuals were higher than those of unvaccinated subjects or those with unknown vaccination history (all *P*-values < 0.05).

**Conclusion:**

Wujin District saw a striking predominance of pertussis cases in school-aged children (5–9 years) in 2024. The antibody levels against pertussis in the healthy population were generally low, especially among adolescents and adults, calling for reinforced active surveillance targeting these age groups.

**Clinical trial number:**

Not applicable.

**Supplementary Information:**

The online version contains supplementary material available at 10.1186/s12879-026-12799-5.

## Background

Pertussis is an acute respiratory infection caused by bordetella pertussis, and primarily transmitted through aerosolized respiratory droplets [1]. It affects people of all ages, with more severe impacts on infants and immunocompromised individuals, and approximately 161,000 children under five die from the disease globally each year [2]. China incorporated the combined diphtheria, tetanus, and whole-cell pertussis vaccine (DTwP) into its national immunization program in 1978. The diphtheria, tetanus, and acellular pertussis vaccine (DTaP) was added in 2007 as an alternative, administered at 3, 4, 5, and 18 months of age. Eventually, the DTaP completely replaced the DTwP by 2012. With the improvement of pertussis vaccination coverage in China, the number of reported cases dropped sharply and stayed consistently minimal. However, the recent resurgence of pertussis poses a serious public health threat [3–5]. China’s pertussis cases have shown a significant upward trend starting in late 2023, with ‌494,321 cases reported in 2024‌—12 times the 2023 figure‌. Meanwhile, the jump in fatalities (31 in 2024 vs. 5 in 2023) has sparked widespread concern [6]. Similarly, pertussis cases in Europe rose dramatically in early 2024 [7], with Denmark’s peak incidence exceeding 330 cases per 100,000, not seen in over two decades [8]. Our previous study found that the incidence of pertussis in Wujin District has been growing progressively since 2017, and a great number of the patients were infants younger than 12 months [9]. Nevertheless, numerous studies have documented that the core reservoirs of infection and vulnerable populations have transitioned from predominantly infants to adolescents and adults [10, 11]. The reported pertussis cases in Wujin District during 2024 ‌hit a decade-high record, given the substantial underdiagnosis and underreporting of pertussis [12], the true transmission pattern of pertussis in Wujin is still unknown. Serological epidemiology is a fundamental tool for appraising intervention effectiveness and assessing persistent infectious disease risks [13]. Hence, this study evaluated the real infection situation and susceptible population of pertussis in Wujin District by analyzing the epidemic characteristics of pertussis in 2024 and the level of pertussis antibodies in healthy populations, providing evidence for precise epidemic prevention and control.

## Methods

### Pertussis surveillance

Pertussis reported cases and demographic information were extracted from the China Disease Control and Prevention Information System, categorized by residential address and date of onset. All cases were laboratory-confirmed, and detailed epidemiological information came from the standardized case investigation questionnaires. Details are presented in the supplementary file.

### Serological survey

A cross-sectional survey on the anti-PT IgG antibody levels of healthy individuals was carried out in Wujin District in 2024. We enrolled 521 study participants and classified them into nine age groups: <1.5, 1.5–3, 4–5, 6–9, 10–14, 15–19, 20–29, 30–39 and ≥ 40 years of age. From each participant, 2 mL of venous blood was drawn, with serum separated and stored at − 20℃ before refrigerated transport for testing. Personal information including age and gender were collected. Immunization histories were retrieved from the Jiangsu Province vaccination integrated service management information system.

### Laboratory assay

All experimental procedures were conducted in the laboratory of Jiangsu Provincial Center for Disease Control and Prevention. Anti-PT IgG levels were quantified using commercial ELISA kits (Zhengzhou Yite Biotechnology Co., Ltd.), and antibody levels were expressed in international units per milliliter (IU/mL). Based on the manufacturer’s instructions, the quantification threshold level for anti-PT IgG was 5 IU/mL. Serum anti-PT IgG concentrations < 20 IU/mL were regarded as negative, and levels ≥ 20 IU/mL were interpreted as positive and indicative of protective immunity. Participants with anti-PT IgG levels ≥ 80 IU/mL and no pertussis vaccination in the past year were identified as having recent infection.

### Statistical analysis

Microsoft Excel 2010 and R Studio 4.4.3 were used for analyses. Pertussis case characteristics in Wujin District (2024) were analyzed with descriptive epidemiology. Continuous variables were summarized as median concentration (MC) and interquartile range (IQR), and between-group differences were assessed using Wilcoxon rank-sum tests. For categorical variables, comparisons of multiple group rates were performed with the *χ*^*2*^ test or Fisher’s exact test. Statistical significance was defined as two-tailed *P* < 0.05.

## Results

### Epidemiological features of pertussis in Wujin District, 2024

28 confirmed pertussis cases were reported in 2024 in Wujin District, comprising 16 males and 12 females, with an annual incidence rate of 2.15 per 100,000 population. A considerable proportion of them were individuals aged between 5 and 9 years old, accounting for 67.86% of the total cases. Next in line was the group of infants under 1 year old (17.86%). In terms of incidence rate, the greatest incidence rate was in the < 1 year cohort (84.02 per 100,000), with the 5–9 year group ranking second (28.70 per 100,000), and the lowest incidence was in the ≥ 10 year old group (0.17 per 100,000). The difference between groups was statistically significant (*χ*^*2*^ = 133.708, *P* < 0.001) (Table 1). 22 cases (78.57%) received the pertussis vaccine in line with the standard immunization schedule. All pediatric cases aged 5–9 years completed the full vaccination course, except for one case with contraindications.

**Table 1:** Age distribution of pertussis cases in Wujin District in 2024

Age (years)	*N*	Percentage (%)	Incidence rate(per 100,000)	*P*-value
< 1	5	17.86	84.02	< 0.001
1–4	2	7.14	5.66	
5–9	19	67.86	28.70	
≥ 10	2	7.14	0.17	

### Basic information on the 2024 pertussis serosurvey in Wujin District

**Fig. 1:**
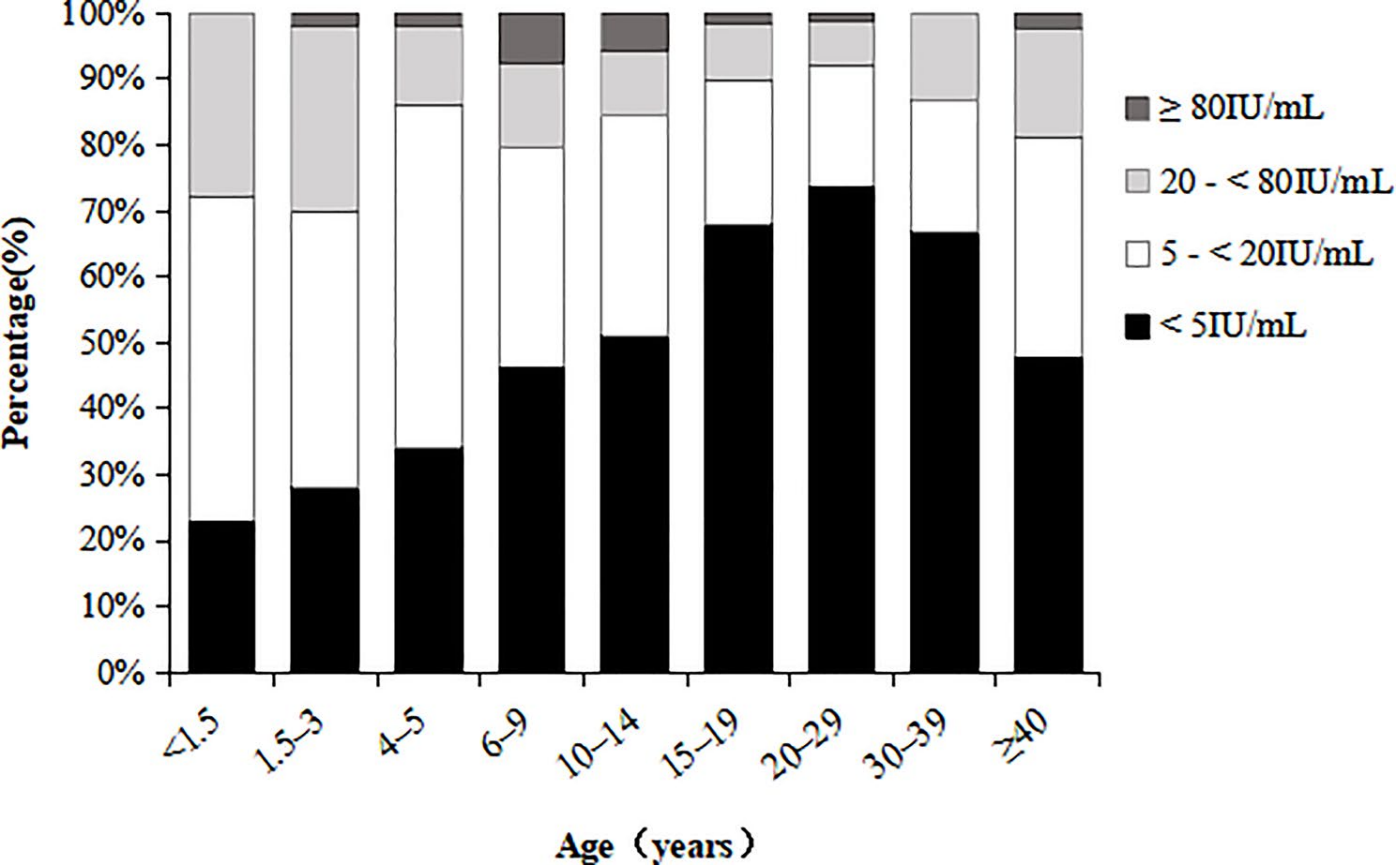
Distribution of PT-IgG levels in the serum of subjects among different age groups

This study involved 521 participants aged from 3 months to 74 years, with a male-to-female ratio of 1.20:1. Nearly half (49.52%) of the subjects showed anti-PT IgG concentrations beneath the quantifiable limit (5 IU/mL), and the proportion of undetectable antibodies gradually increased with age in the < 30-year cohort (trend *χ*^*2*^ = 55.512, *P* < 0.001). The average anti-PT IgG seropositivity rate‌ and MC‌ level were ‌17.27% (90/521)‌ and ‌5.17 IU/mL‌, respectively. Twelve out of 90 participants with protective titers (≥ 20 IU/mL) had antibody levels of ≥ 80 IU/mL. After excluding one case with pertussis vaccination in the preceding 12 months, the recent infection rate was 2.11% (Fig. 1; Table 2).

**Table 2:** Serological epidemiological characteristics of pertussis in healthy population in Wujin District in 2024

Characteristic	*N*	MC (IU/mL, IQR)	*P*-value	Positive rate (%)	*P*-value	Infection rate (%)	*P*-value
Gender							
Male	284	4.54(0.59–14.71)	0.514	57(20.07)	0.065	5(1.76)	0.542
Female	237	2.70(0.61–13.71)		33(13.92)		6(2.53)	
Age group (years)							
< 1.5	61	11.54(5.61–22.09)	< 0.001	17(27.87)	0.017	0(0.00)	0.129
1.5–3	50	11.66(3.78–22.64)		15(30.00)		0(0.00)	
4–5	50	5.85(2.95–11.14)		7(14.00)		1(2.00)	
6–9	39	6.19(0.75–16.28)		8(20.51)		3(7.69)	
10–14	51	4.99(2.25–13.52)		8(15.69)		3(5.88)	
15–19	59	2.88(0.26–7.92)		6(10.17)		1(1.69)	
20–29	76	0.19(0.00-6.13)		6(7.89)		1(1.32)	
30–39	45	1.29(0.10–9.14)		6(13.33)		0(0.00)	
≥ 40	90	5.71(1.17–13.96)		17(18.89)		2(2.22)	
Immunization history							
Vaccinees	260	7.97(2.97–16.11)	< 0.001	55(21.15)	0.019	7(2.69)	0.357
Nonvaccinees/unknown	261	2.25(0.09–10.38)		35(13.41)		4(1.53)	
Total	521	5.17(0.59–14.22)		90(17.27)		11 (2.11)	

### Seroepidemiological characteristics of pertussis in the healthy population of Wujin

Anti-PT IgG seropositivity rates and concentrations did not differ significantly by gender (all *P* > 0.05), while varied markedly across age groups (all *P* < 0.05). The anti-PT IgG antibody positivity rate was highest in children aged 1.5–3 years (30.00%), then it dropped precipitously to 14.00% in the 4-5-year-old group and subsequently climbed to 20.51% in the 6-9-year-old group. Afterwards, the positive antibody rate continued to decrease with age, reaching its lowest point (7.89%) between 20 and 29 years old. The seroprevalence turned up after 30 years old, yet it remained at relatively low levels. The trend of anti-PT IgG antibody concentration changing with age was consistent with the antibody positivity rate. Vaccinated individuals had higher seropositivity and concentration compared to unvaccinated subjects or those whose vaccination history was unknown (all *P* < 0.05). Recent infection rates were most pronounced in the 6–9 years age group (7.69%), followed by 10-14-year-olds (5.88%) (Table 2).

## Discussion

The reported incidence of pertussis in Wujin District reached 2.15 per 100,000 population in 2024, surpassing all recorded rates since 2012 [9]. This rate exceeded that of Weihai city (1.74 per 100,000 in 2023) [14] while being below those for Nanjing city (2.62 per 100,000 in 2022) [15], the 2023 national average (2.92 per 100,000) [16], and Russia (36.29 per 100,000) [17]. In 2024, both the proportion and incidence of pertussis in children aged 5–9 years in Wujin have risen markedly relative to‌ the average of the past ten years[9]. Despite higher incidence in infants aged < 1 year, children aged 5–9 years made up 67.86% of pertussis cases, in contrast to 17.86% in infants. Similar to our results, two-thirds of the 15 pertussis public health emergencies in China in 2022 were clustered in schools, with 2 taking place in kindergartens and 8 in primary schools [18]. Besides, the pertussis surveillance data of Beijing Children’s Hospital highlighted a dramatic surge in the group of school-aged children (≥ 6 years), with their proportion rising from 1.4% in 2019 to 36.5% in 2023, suggesting a shift in the pertussis burden to schoolchildren, which could be a major driver of the disease’s spread [19]. Factors contributing to the recurrence of pertussis and changes in age-specific incidence patterns comprise declining vaccine-induced protection, inadequate supplementary immunization, refined diagnostic approaches, and expanding prevalence of macrolide-resistant Bordetella pertussis [20, 21].

Due to misdiagnosis and insufficient surveillance systems, there is a high probability that the actual incidence rate has been underestimated. Therefore, we implemented the anti-PT IgG antibody survey to explore the pertussis immunity in healthy residents in Wujin. The results indicated that the overall anti-PT IgG antibody positivity rate in the healthy population of Wujin District was 17.27%, higher than the serological findings in Jiangsu Province (14.76%, 2018) [22] and Henan Province (12.10%, 2022–2023) [23], but lower than those in Zhejiang Province (29.80%, 2020) [24] and Chonburi Province of Thailand (57.4%, 2022–2023) [25]. Pertussis is a highly contagious disease with a basic regeneration number (R_0_) of 12–17 [26], requiring 91.67–94.12% population immunity to interrupt transmission. Regrettably, the positive rate of pertussis antibody among healthy individuals in Wujin District significantly lags behind this required level, which means a large number of people are prone to infection. According to the prior immunization strategy, the lack of DTaP revaccination after the age of 2, combined with antibody decay, likely constitutes the principal reason for the low pertussis seroprevalence rate in the healthy population of Wujin District. In this study, children aged 1.5–3 years demonstrated superior antibody seropositivity and concentrations as opposed to other age groups. Conversely, these values fell to less than half in children aged 4–5 years, showing both a robust post-booster immune response and a rapid decline in anti-PT IgG levels within four years after completing the full-course immunization. This aligns with the outcome from other investigations‌, which reported that the protective efficacy of acellular pertussis vaccines declines 2–3 years after booster vaccination, with an immune persistence of approximately 5 years [27, 28]. The rebound in the antibody levels of 6-9-year-old group, alongside the notable recent infection rate (7.69%), suggests ‌active pertussis transmission [19]. This was echoed by the above‌-mentioned heightened incidence of pertussis cases in children aged 5–9 years, and underscored that pertussis antibody surveillance could serve as an early warning system for populations at elevated risk. The variation of pertussis antibodies by age mirrored the anti-PT IgG seroprevalence patterns found in Chinese population meta-analyses [29]. Obviously, the four dose immunization program was unable to provide adequate protection for school-age children. Preschool booster vaccination with pertussis-containing vaccines has been proven effective in reducing pertussis incidence in various countries [30]. China introduced a ‌5 dose DTaP immunization strategy‌ beginning on January 1, 2025, with vaccinations given at ‌2 months, 4 months, 6 months, 18 months, and 6 years of age. The revised pertussis vaccination policy in China, now compliant with international standards, is expected to enhance vaccine efficacy and disease control efforts.

It is noteworthy that even though the anti-PT IgG antibody levels in individuals aged 10 years and older (particularly the 20-29-year-old childbearing age group) were rather low, only 2 pertussis cases in this age group were reported in 2024, less than 10% of the total cases. This discrepancy could be attributed to a large proportion of cases presenting as mild or asymptomatic, leading to underreporting, reflecting potential underestimation of pertussis incidence in adolescents and adults [31]. In a multicenter Asian study, recent pertussis infection was present in roughly 5% of adolescents (10–18 years), regardless of vaccination conditions [32]. There has been literature identifying pertussis outbreaks in households where adults were the primary case, causing 43% of secondary cases [33]. Adolescent and adult pertussis cases, as family members, are the main source of infection for newborns and infants [34]. Strengthening pertussis surveillance in these populations is essential for infant disease prevention. Moreover, it is urgent to improve the diagnostic infrastructure at rural medical institutions, as only one out of 17 township health centers in Wujin District has the PCR testing capacity for pertussis. The antibody levels of those aged 40 or older have gone up appreciably, which may be linked to the immunity generated by natural infection [24].‌‌‌

A key advantage of our study is its comprehensive coverage of the entire age spectrum with well-balanced age and gender distribution, thus ensuring good representativeness. There are also certain limitations to this study. First, since vaccine-induced and infection-derived antibodies are indistinguishable in infants and children, precise estimation of pertussis infection rates in these age groups is not possible. Second, constrained by the information system, we could not access the immunization registers of people aged over 15. Considering that there is no DTaP vaccination program for adolescents in China, their anti-PT antibodies are probably induced by infection [35]. Finally, the correlation between antibody levels and protective efficacy is incomplete [36], and cell-mediated immunity can last for several years [37].

In summary, the majority of pertussis cases in Wujin District in 2024 occurred among school-age children aged 5 to 9 years old. The population-based serosurvey revealed universally low anti-PT IgG seropositivity rates across all age groups (particularly ≥ 10 years) in Wujin’s healthy residents, indicating ‌prevalent susceptibility. The effectiveness of the adjusted DTaP vaccination schedule, as well as whether the high-risk groups may transfer toward teenagers and adults in the future, requires ongoing monitoring and analysis.

## Supplementary Information

Below is the link to the electronic supplementary material.

Supplementary Material 1

## Data Availability

Data will be provided as required.‌.
