# Supplementary material for: Pertussis epidemiology and seroprevalence in Wujin District, Changzhou, China, 2024
Source: BMC Infect Dis. 2026 Feb 12;26:588. doi: 10.1186/s12879-026-12799-5 (PMC13005502; doi:10.1186/s12879-026-12799-5)
Supplement: Supplementary file 1 — Supplementary Material 1 [file 12879_2026_12799_MOESM1_ESM.doc]

****Pertussis Case Investigation Form****
****Case Code**** □□□□□□□□□□□□□

### ****I. Basic Information****

1. ****Name (Required)****: ________
2. ****Gender (Required)****: □ Male; □ Female
3. ****Ethnicity (Required)****: ______
4. ****Current Residential Address (Detailed)****: ____City_________District (County)______Street (Township)___________Neighborhood Committee (Village)
5. ****Date of Birth (Required)****: ____Year___Month___Day
6. ****Contact Number (Required)****: __________
7. ****Occupation of the Patient (Required)****:
   □ Preschool children; □ Scattered children; □ Students; □ Teachers (primary, secondary, tertiary education); □ Nursery staff and nannies; □ Catering and food industry; □ Commercial services; □ Medical staff; □ Workers; □ Farmers; □ Herdsmen; □ Fishermen (fishing vessel crew); □ Cadres and office staff; □ Retirees; □ Housework and unemployed; □ Others: __________
8. ****Past Medical History (Required)****:
   □ Asthma; □ Emphysema; □ Chronic bronchitis; □ Immunodeficiency; □ AIDS; □ Tuberculosis; □ Malnutrition; □ Other diseases: ________; □ None

### ****II. Clinical Manifestations and Treatment****

1. ****Date of First Symptom Onset (Required)****: ____Year___Month___Day
2. ****Diagnosis Date (Required)****: ____Year___Month___Day
3. ****Clinical Symptoms at This Visit (Required)****:
   3.1 Fever: □<38℃; □38~38.5℃; □38.6-39℃; □＞39℃; □ No fever; □ Unknown
   3.2 Cough: □ Yes, onset date: ____Year___Month___Day; □ No; □ Unknown
   3.3 Paroxysmal or spasmodic cough: □ Yes, onset date: ____Year___Month___Day; □ No; □ Unknown
   3.4 Inspiratory stridor (high-pitched whoop): □ Yes; □ No; □ Unknown
   3.5 Vomiting: □ Yes; □ No; □ Unknown
   3.6 Cyanosis of lips: □ Yes; □ No; □ Unknown
   3.7 Asphyxia: □ Yes; □ No; □ Unknown
   3.8 Other symptoms: ___________
4. ****Complications (Required)****:
   4.1 Pneumonia: □ Yes; □ No; □ Unknown
   4.2 Encephalopathy: □ Yes; □ No; □ Unknown
   4.3 Other complications: □ Yes: _________; □ No; □ Unknown
5. ****Hospitalization (Required)****: □ Yes; □ No; □ Unknown
   5.1 Was the direct cause of hospitalization pertussis? □ Yes; □ No, reason for hospitalization: ______________

### ****III. Laboratory Tests****

1. ****Laboratory test items (Required, multiple choices allowed)****:
   □ Blood routine; □ Chest X-ray; □ Bacterial culture; □ PCR test; □ Serum antibody test; □ Not performed
2. ****Test results****:
   2.1 Blood routine results:
   White blood cell count: ; Lymphocytes: %; Neutrophils: %
   Specimen collection date: ____Year___Month___Day
   2.2 Hospital X-ray (fluoroscopy) showed pneumonia: □ Yes □ No □ Not done
   2.3 Bacterial culture: □ Positive; □ Negative; Specimen collection date: ____Year___Month___Day
   2.4 PCR test: □ Positive; □ Negative; Specimen collection date: ____Year___Month___Day
   2.5 Serum antibody test:
   First sample: ; Specimen collection date: ____Year___Month___Day
   Second sample: ; Specimen collection date: ____Year___Month___Day
   2.6 Metagenomic/multiple pathogen test: □ Positive; □ Negative
   Specimen collection date: ____Year___Month___Day

### ****IV. Epidemiological Investigation Information****

1. ****Contact with suspected or confirmed pertussis cases? (Required)****: □ Yes; □ No; □ Unknown
   1.1 Contact mode: □ Household contact; □ Same workplace; □ Same class; □ Others: _______
2. ****Study, work, or live in a collective unit (e.g., school, kindergarten, factory, etc.)? (Required)****:
   □ Yes, collective unit name: ____; □ No; □ Unknown
3. ****Possible source of infection for this case (Required)****:
   □ Kindergarten; □ School (primary, secondary, tertiary); □ Family; □ Hospital infection; □ Workplace; □ Others: _____; □ Unknown
4. ****Vaccination history with pertussis-containing vaccines****:
   4.1 Has pertussis-containing vaccine been administered?
   □ Yes, fill in the table below; □ No, skip to 6.; □ Unknown, end the investigation

| **Dose** | **Vaccination Date** | **Vaccine Type ^a^** | **Immunization History Source ^b^** |
| --- | --- | --- | --- |
| 1 |  |  |  |
| 2 |  |  |  |
| 3 |  |  |  |
| 4 |  |  |  |
| 5 |  |  |  |

a: ① Acellular DTP vaccine; ② Whole-cell DTP vaccine; ③ DTP-Hib combined vaccine (quadrivalent vaccine); ④ DTaP-IPV/Hib combined vaccine (pentavalent vaccine);
b: ① Vaccination certificate; ② Information system; ③ Parental recall
5. ****If not vaccinated or not completed 4 doses, reason for non-vaccination (Required)****:
□ Vaccination contraindication; □ Delayed due to illness; □ Other non-illness reasons for missed vaccination; □ Parental refusal; □ Not yet of vaccination age; □ High vaccination cost; □ Unaware of vaccination need; □ Unknown
